# Worse cardiovascular and renal outcome in male SLE patients

**DOI:** 10.1038/s41598-023-45171-7

**Published:** 2023-10-30

**Authors:** Jelena Mihailovic, Camillo Ribi, Carlo Chizzolini, Marten Trendelenburg, Johannes Von Kempis, Suzan Dahdal, Uyen Huynh-Do, The Swiss Systemic Lupus Erythematosus Cohort Study Group (SSCS), Denis Comte, Ute Eisenberger, Thomas Hauser, Pascale Roux-Lombard, Andrea Rubbert-Roth, Urs Steiner

**Affiliations:** 1 Department of Nephrology and Hypertension Inselspital, University Hospital Bern, Freiburgstrasse 18, 3010 Bern, Switzerland; 2 Division of Clinical Immunology and Allergy, University Hospital Lausanne, Lausanne, Switzerland; 3 Pathology and Immunology, School of Medicine, Geneva University, Geneva, Switzerland; 4 Division of Internal Medicine and Clinical Immunology Laboratory, Department of Biomedicine, University Hospital Basel, Basel, Switzerland; 5 Division of Rheumatology and Immunology, Department of Internal Medicine, Kantonsspital St Gallen, St Gallen, Switzerland; 6 Division of Internal Medicine, University Hospital Basel, Basel, Switzerland; 7 Department of Nephrology, University Hospital Essen, Essen, Germany; 8 IZZ Immunologie-Zentrum Zürich, Zurich, Switzerland; 9 Department of Immunology and Allergy, Geneva University Hospital, Geneva, Switzerland; 10 Department of Dermatology, Kantonsspital St Gallen, St Gallen, Switzerland; 11 Department of Immunology, University Hospital of Zurich, Zurich, Switzerland

**Keywords:** Cardiology, Diseases, Nephrology, Rheumatology

## Abstract

Systemic lupus erythematosus (SLE) in males is rare and poorly understood. Thus, still little is known about sex differences in SLE. We set out to identify sex differences regarding clinical manifestations as well as renal and cardiovascular outcomes of SLE. We analyzed patient data from the Swiss SLE Cohort Study. Cumulative clinical manifestations according to the updated American College of Rheumatology criteria were recorded at inclusion. Cardiovascular events were recorded within Systemic Lupus International Collaborating Clinics/American College of Rheumatology Damage Index (SLICC-SDI). Renal failure was defined as eGFR < 15 ml/min/1.73 m^2^, initiation of renal replacement therapy or doubling of serum creatinine which were all assessed yearly or documented as end stage renal disease in SLICC-SDI. Risk differences were calculated using logistic regression and cox regression models. We analyzed 93 men and 529 women with a median follow up time of 2 years. Males were significantly older at diagnosis (44.4 versus 33.1 years, p < 0.001) and had less often arthritis (57% versus 74%, p = 0.001) and dermatological disorders (61% versus 76%, p < 0.01). In multivariate analysis female sex remained a significantly associated with arthritis and dermatological disorders. In multivariate analysis men had a significantly higher hazard ratio of 2.3 for renal failure (95% confidence interval (95%-CI) 1.1–5.2, p < 0.04). Total SLICC-SDI Score was comparable. Men had significantly more coronary artery disease (CAD) (17% versus 4%, p < 0.001) and myocardial infarction (10% versus 2%, p < 0.01). In multivariate analysis, male sex remained a significant risk factor for CAD (odds ratio (OR) 5.6, 95%-CI 2.3–13.7, p < 0.001) and myocardial infarction (OR 8.3, 95%-CI 2.1–32.6, p = 0.002). This first sex study in a western European population demonstrates significant sex differences in SLE. Male sex is a risk factor for cardiovascular events and renal failure in SLE. Potential etiological pathomechanisms such as hormonal or X-chromosomal factors remain to be further investigated.

## Introduction

Systemic lupus erythematosus (SLE) is a chronic autoimmune disease with a wide range of clinical manifestations and a potentially life-threatening outcome. As in other autoimmune diseases, women are much more affected than men are. An European study reported a female to male ratio of 10:1^1^. As a consequence, male SLE is still poorly understood.

In the past decades, multiple studies showed striking evidence for sex differences which in turn aroused the interest in sex specialized medicine. Recent studies reported a higher mortality in males than females in systemic sclerosis, another autoimmune disease^2,3^. Thus, one could assume similar findings in SLE, and indeed, already in 1981 Wallace et al. reported a higher mortality in men with SLE than in women^4^. The more recent large LUMINA study showed a poorer long term prognosis with accelerated development of damage in men compared to women^5^. In the study of Roman et al. accelerated atherosclerosis was found to occur prematurely and independent of traditional risk factors for cardiovascular disease in SLE^6^. Cardiovascular disease is one of the main causes of death in SLE and indeed the LUMINA study identified male sex as a risk factor for cardiovascular events in patients with SLE^7^. Tan et al. showed in a large American cohort a higher rate of renal failure and end stage renal disease (ESRD) in males^8^. However, multiple other studies do not confirm these findings, especially regarding renal involvement and mortality^9^. For example, Renau et al. observed an increase in renal failure and death in females with SLE^10^ and Voulgari et al. showed no overall increase in renal involvement in men^11^. Furthermore, ethnic background and socioeconomic status are known to influence the presentation and disease course of SLE^12^. The ethnic background of patients enrolled in the LUMINA study consists of only 28% Caucasians which differs widely from reported ethnic backgrounds of western European SLE population^13^. This rises the need for an investigation of sex differences in SLE in a western European cohort. Likewise, the American cohort by Tan et al. includes only 60% Caucasians.

Thus, differences between male and female SLE described in the literature remain controversial and further investigation are needed. This motivated us to study in more detail sex differences in SLE using the first Swiss SLE cohort derived from different tertiary, secondary and primary care centers and lasting over 10 years. We took advantage of this prospective, multidisciplinary Swiss systemic lupus erythematosus cohort study (SSCS)^14^ to address that question and compared SLE manifestations and the renal and cardiovascular outcome of male versus female patients.

## Methods

All patients in this study were included in the Swiss SLE Cohort Study. This nationwide prospective cohort was established in 2007 as a collaboration between tertiary, secondary and primary care centers encompassing various medical specialties in Switzerland^14^.

### Patients and data

Patients at least 16 years old with diagnosed SLE according to the updated American College of Rheumatology (ACR) criteria^15,16^ and informed consent were continuously included into the cohort between 2007 and 2017 by their treating doctor. In the case of two patients, who were 16.5 and 17.9 years old at the time of inclusion, informed consent was obtained from the parents or the legal guardians of the patient. Patient data such as age, sex, ethnic background, family history of SLE, date of first SLE manifestation and date of diagnosis was collected at inclusion. The presence of all cumulative clinical manifestations defined by the updated ACR classification criteria of SLE prior to inclusion were reported. A follow up was conducted yearly and at disease flares by the patient’s treating doctor. At inclusion and at each follow up laboratory values as serum haemoglobin, thrombocytes, creatinine, erythrocytes sedimentation rate were measured. Cardiovascular risk factors such as hypertension, diabetes mellitus type 2, hyperlipidemia, coronary heart disease and cerebrovascular disease were collected at patient’s inclusion to cohort. Additionally at inclusion and every follow up medication, disease activity, need for renal replacement treatment and deaths of the patients were reported. Disease activity was measured with Systemic Lupus Erythematosus Disease Activity Index score with the Safety of Estrogens in Lupus Erythematosus modification (SELENA SLEDAI) score and physicians global assessment (PGA) score^14^. At least once during the follow up period the Systemic Lupus International Collaborating Clinics/American College of Rheumatology Damage Index (SLICC-SDI) was assessed for patients with over six months of disease duration. The SELENA SLEDAI^17^ as well as the SLICC-SDI^18^ are standardized scores to quantify disease activity of SLE or respectively the cumulative and irreversible organ damage and make comparisons between the patients in studies possible.

The estimated glomerular filtration rate (eGFR) was calculated by chronic kidney disease epidemiology collaboration equation (CKD-EPI)^19^ based on serum creatinine values. The decision to perform a renal biopsy was left to the treating physician according to clinical practice. The performed renal biopsies were reported at patient’s inclusion or at the next follow up. We analyzed the most recent available biopsy result of a patient. Lupus nephritis was either classified according to International Society of Nephrology and Renal Pathology Society (ISN/RPS)^20^ or to World Health Organization (WHO) 1982 modified classification^21^. The medication was categorized in three groups: antimalarial drugs, systemic glucocorticoids and immunosuppressive agents other than glucocorticoids. We analyzed how many patients used one of the medication at least once during follow up period or at baseline.

### Outcomes

We investigated the difference in renal outcome between the sexes. Renal failure was defined as eGFR < 15 ml/min/1.73 m^2^, initiation of renal replacement therapy, documented end stage renal disease in SLICC-SDI or doubling of serum creatinine. In order to adjust for confounders, we performed multivariate cox regression for renal failure. Furthermore, we analyzed the overall damage caused by SLE and the occurrence of cardiovascular events with the information provided by SLICC-SDI. A multivariate analysis was performed to control the sex differences in cardiovascular outcomes for confounders.

### Statistical analysis

Data are presented as absolute numbers with percentages for categorical variables, as medians with 25%- and 75%-quartiles for not normally distributed continuous variables or as means with standard deviation for normally distributed variables. Comparison between two groups was assessed by Mann–Whitney U test for continuous variables and chi-square test or fisher’s exact test for categorical variables. In the presented Tables 1 and 2 only univariate comparisons were displayed.

**Table 1:** Baseline characteristics of the study population.

Demographics	Women	Men	p-value^a^
Number of patients	529 (85%)	93 (15%)	
Age at baseline (years)	42.9 (32.0, 53.3)	48.2 (36.2, 65.9)	**0.002**
Ethnic background			
Caucasian	416 (81%)	73 (82%)	0.995
African	36 (7%)	6 (7%)	
Asian	44 (9%)	7 (8%)	
Other	19 (4%)	3 (3%)	
First degree relatives with SLE	58 (13%)	8 (11%)	0.709
SLE characteristics			
Age at diagnosis (years)	33.1 (24.1, 45.6)	44.4 (28.7, 57.0)	** < 0.001**
Time to diagnosis (years)	0.3 (0.0, 2.0)	0.3 (0.0, 1.7)	0.956
Disease duration (years)	3.4 (0.8, 10.2)	2.2 (0.4, 7.2)	**0.018**
Follow up time (years)	2.0 (0.0, 4.9)	2.0 (0.0, 4.9)	0.815
Laboratory assessment at baseline			
Haemoglobin (g/l)*	123 ± 22	134 ± 21	** < 0.001**
Thrombocytes (G/L)*	243 ± 89	226 ± 88	**0.011**
Serum creatinine (µmol/l)	67 (59, 79)	86 (73, 99)	** < 0.001**
eGFR (ml/min/1.73 m^2^)	94 (74, 111)	93 (70, 111)	0.412
Cardiovascular risk factors at baseline			
Hypertension	22 (4%)	14 (15%)	** < 0.001**
Diabetes mellitus type 2	21 (4%)	7 (8%)	0.127
Hyperlipidemia	111 (21%)	27 (29%)	0.085
Coronary heart disease	18 (3%)	12 (13%)	** < 0.001**
Cerebrovascular disease	39 (7%)	13 (14%)	**0.034**
Medication^b^			
Antimalarial medication	414 (79%)	66 (73%)	0.217
Immunosuppressant agents	302 (57%)	65 (71%)	**0.021**
Oral corticosteroids	331 (63%)	70 (76%)	**0.018**

Values were presented as medians with 25% and 75% quartile in brackets for continuous variables or as absolute values with percentages for categorical variables.

*Normally distributed continuous variables were presented as mean ± standard deviation.

^a^P-values < 0.05 were considered statistically significant and written in bold.

^b^Use of antimalarial medication, oral corticosteroids or immunosuppressants other than corticosteroids at baseline and/or at least once during the follow up period.

**Table 2:** Damage accrual and disease activity.

	Women	Men	p-value^a^
Total patients	475	83	
Age at SLICC-SDI (years)	45.7 (35.0, 57.1)	52.4 (40.7, 68.5)	**0.001**
Disease duration at SLICC-SDI (years)	6.9 (3.0, 13.6)	5.5 (2.0, 10.7)	**0.026**
Time between inclusion and SLICC-SDI (years)	2.0 (0.0, 4.9)	1.4 (0.0, 4.6)	NS
SLICC-SDI			
Score (points)	0 (0, 2)	1 (0, 3)	0.072
Cerebrovascular insult	27 (6%)	6 (7%)	0.789
Any renal complication	52 (11%)	10 (12%)	0.709
Chronic kidney disease	44 (9%)	9 (11%)	0.051
Proteinuria	13 (3%)	2 (2%)	0.658
ESRD	13 (27%)	3 (4%)	0.718
Any cardiac complication	57 (12%)	20 (24%)	**0.006**
Coronary vascular disease	20 (4%)	14 (17%)	** < 0.001**
Myocardial infarctus	11 (2%)	8 (10%)	**0.003**
Cardiomyopathy	21 (4%)	5 (6%)	0.568
Valvular disease	26 (5%)	7 (8%)	0.310
Chronic pericarditis	7 (1%)	0 (0%)	0.601
Any peripher vascular complication	13 (3%)	2 (2%)	1.0
Claudication	6 (1%)	2 (2%)	0.339
Minor tissue loss	7 (1%)	0 (0%)	0.601
Significant tissue loss	3 (1%)	0 (0%)	1.0
Disease activity			
SELENA SLEDAI at baseline (points)	4 (2, 8)	4 (2, 9)	0.496
PGA at baseline (points)			
Inactive	243 (46%)	41 (44%)	0.484
Moderately active	184 (35%)	35 (38%)	
Active	87 (16%)	12 (13%)	
Very active	15 (3%)	5 (5%)	
ESR (mm/h) at baseline	14 (7, 33)	16 (7, 35)	0.861

Values were presented as medians with 25% and 75% quartile in brackets for continuous variables or as absolute values with percentages for categorical variables.

^a^P-values < 0.05 were considered statistically significant and written in bold.

For categorical outcome variables such as SLE manifestations and cardiovascular outcomes a multivariate analysis was performed using multiple logistic regression. We included in the logistic regression models independed variables, which had a p-value < 0.05 in the univariate comparison with the outcome variable. In a second step we checked for interactions between the significant, independent variables. The different models were compared by akaike information criterion (AIC) and the model with the lowest AIC was chosen.

We initially included sex, age at inclusion, disease duration, time from SLE manifestation to diagnosis, ethnic background, medication used during follow up period, eGFR at inclusion and SELENA-SLEDAI Score at inclusion in the multivariate cox regression model. The least significant variables were then removed step by step from the model. Finally, a model including sex, age at inclusion, time from disease manifestations until diagnosis and eGFR at inclusion as independent variables remained. Renal failure curves were developed with cox regression.

Overall mortality during the follow up period was analyzed using kaplan meier survival curves and compared with log rank test. Additionally, age adjusted mortality was analyzed using cox regression which included sex and age at inclusion as independent variables.

In all cox regression models patients were included at inclusion to the cohort and censored at their last follow up visit. We assessed all cox proportional models for violation of proportional hazards assumption. A p-value < 0.05 was considered statistically significant. Statistical analysis was performed with IBM SPSS Statistics 23.0 statistical software package (SPSS Inc, Chicago, Illinois, USA).

### Ethics approval and consent to participate

All data was collected from the Swiss SLE Cohort Study which was approved by the ethics review boards of all participating institutions (i.e. Commission cantonale d'éthique de la recherche sur l'être humain du Canton de Vaud CER-VD, kantonale Ethikkommission Bern, Ethikkommission Nordwest- und Zentralschweiz EKNZ, commission cantonale d’éthique de la recherche Genève CCER, Ethikkommission Ostschweiz EKOS, kantonale Ethikkommission Zürich) and all patients gave written informed consent. In the case of two patients, who were 16.5 and 17.9 years old at the time of inclusion, informed consent was obtained from the parents or the legal guardians of the patient. The study was conducted according to the guidelines of the Declaration of Helsinki.

## Results

### Patients’ baseline characteristics

We analyzed a total of 622 patients in our cohort, of which 529 (85%) were female and 93 (15%) were male. The female to male ratio was 5.7:1. The majority of patients were Caucasian (81% of females and 82% of males) and the ethnic background was comparable between males and females.

The median age at diagnosis was 33.1 years in women and 44.4 years in men, the difference was significant with a p-value < 0.001. Men were significantly older at inclusion than women (48.2 versus 42.9 years, p = 0.002) and had a significantly shorter median disease duration at inclusion (2.2 versus 3.4 years, p = 0.018). The median time between onset of symptoms and SLE diagnosis was 0.3 years in both sexes. In total 49 patients (8%) were lost to follow up of which 4 were males (4% of all males) and 45 were females (9%).

Medication used during follow up period differed between the two groups: Significantly more males were treated at least once during the follow up period with immunosuppressant agents and oral corticosteroids than females (Table 1). The difference in the use of antimalarial medication was not significant.

### Clinical and immunological manifestation

At inclusion, men had a statistically significant lower median number of cumulative ACR criteria than women (4 versus 5 points, p-value = 0.007). Women had significantly higher prevalence of dermatological manifestation, 403 women (76%) versus 57 men (61%) (p = 0.005). Regarding the individual dermatological manifestation, only the difference in photosensitivity was significant. Arthritis was more common in women than in men, 392 (74%) and 53 (57%) respectively (p = 0.001). Women had a higher prevalence of psychosis with 34 women (6%) versus one man (1%) (p = 0.047). There were no significant differences in other clinical or immunological manifestations (supplementary file 1).

In multivariate analysis for arthritis we included sex, anti-Sm antibodies, anti-dsDNA antibodies, eGFR at inclusion, SELENA SLEDAI Score at inclusion, oral corticosteroids at inclusion, age at diagnosis and disease duration at inclusion in the model. Sex, anti-Sm antibodies, oral corticosteroids, eGFR and disease duration were all significantly associated with arthritis. Male sex had an odds ratio (OR) of 0.31 for arthritis with a 95% confidence interval (95%-CI) 0.16–0.59, p < 0,001. After inclusion of an interaction factor between sex and disease duration, the effect of disease duration was reduced but remained significant (supplementary Table 2).

In multivariate analysis for dermatological manifestations, we included sex, disease duration at inclusion, PGA at inclusion and anti-Sm antibodies. Only sex and disease duration were significant. When including only sex and disease duration in the model, only male sex was significant with an OR of 0.51 (95%-CI 0.32–0.82, p = 0.005) (supplementary Table 3). After inclusion of the interaction factor between sex and disease duration, the effect of sex was not significant any more, but the overall multivariate model had a higher AIC showing no overall improvement of the model.

In the multivariate model for photosensitivity, we included sex, ethnic background, age at diagnosis, disease duration at inclusion, PGA at inclusion, anti-dsDNA antibodies and antimalarial medication at inclusion. Sex, ethnic background, anti-dsDNA antibodies, PGA, antimalarial medication and disease duration were all significant. Male sex had an OR of 0.41 (95%-CI 0.24–0.70, p = 0.001) (supplementary Table 4). The interaction factor between sex and disease duration did not significantly change the results nor was the overall model improved.

### Disease activity

There was no significant difference in disease activity at baseline between the sexes. The SELENA-SLEDAI Score, PGA and erythrocyte sedimentation rate (ESR) were all similar (Table 2).

### Overall damage outcome

We compared the most recent SLICC-SDI Score between the two groups which was available for 475 women and 83 men. In these two groups men were older at the time of SLICC-SDI Score (52.4 versus 45.7 years, p = 0.001). Women had a significantly higher disease duration at the time of the SLICC-SDI (6.9 versus 5.5 years, p = 0.026). Men tended to have higher SLICC-SDI Score (Table 2).

### Lupus nephritis

Renal disease occurred in 39 men (42%) and in 197 women (37%). This difference was not significant. Results from renal biopsies were available for 20 men and 89 women. The most common type of lupus nephritis without regard to classification system was class IV in 11 men (55%) and 35 women (39%), followed by class III in 6 men (30%) and 18 women (20%). The overall distribution of lupus nephritis classes was similar between sexes.

### Renal outcome

**Figure 1:**
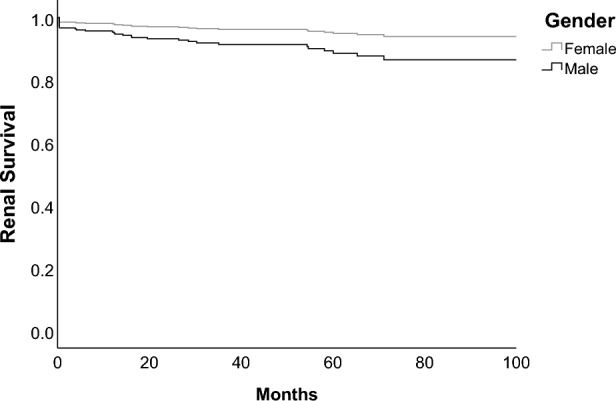
Cox regression survival curve for renal failure in men and women. Renal failure was defined as eGFR < 15 ml/min/1.73 m^2^, initiation of renal replacement therapy, documented end stage renal disease in SLICC-SDI or doubling of serum creatinine. Regression model included sex, age at inclusion, time to diagnosis from disease manifestation and eGFR at inclusion.

We compared the incidence of renal failure between sexes using cox regression model. Data of renal outcome was available for in total 512 patients (430 females and 82 males), 38 of them had renal failure. We included sex, age at inclusion, time from disease manifestation to diagnosis and eGFR at inclusion in the cox regression model (Table 3). Male sex had a significantly increased hazard ratio of 2.3 with a 95%-CI 1.1–5.2 and a p-value of 0.036 (Fig. 1). We performed additionally a cox regression model including sex, eGFR at baseline and cardiovascular risk factors such as diabetes mellitus type 2, hypertension, hyperlipidemia and coronary heart disease at baseline (supplementary file 5). In this model male sex, as well as cardiovascular risk factors, did not have a significantly increased hazard ratio. However, this overall model had a higher AIC compared to the model described above.

**Table 3:** Cox regression model for renal failure.

	Unadjusted model		Adjusted model		
	HR	95%-CI	HR	95%-CI	p-value
Sex	1.769	0.839–3.727	2.335	1.055–5.165	0.036
Age at inclusion	1.003	0.982–1.023	0.973	0.950–0.996	0.021
Time to diagnosis	1.011	0.969–1.056	1.035	0.994–1.077	0.100
eGFR at inclusion	0.959	0.949–0.969	0.959	0.948–0.969	< 0.001
Overall model					
AIC					381.995
p-value					< 0.001

Table of estimates of cox regression model for renal failure. Unadjusted model presents results from a univariate cox regression, adjusted model presents results from the multivariate cox regression including all shown variables. Sex (0 = female, 1 = male), age in years at baseline and time from SLE manifestation to SLE diagnosis in years were included in the model. HR = hazard ratio, 95%-CI = 95%-confidence interval.

### Cardiovascular outcome

In the most recent SLICC-SDI Score men had significantly higher rates of coronary arterial disease (CAD) and myocardial infarction. 14 men (17%) versus 20 women (4%) had CAD (p < 0.001). Eight men (10%) versus 11 women (2%) had myocardial infarction (p = 0.003). Cerebrovascular, peripheral vascular and cardiac complications were not significantly different (Table 3). The SLICC SDI Score was available of males 83 and females 475 (Table 2).

We performed a multiple logistic regression for CAD in which we included sex, age at time of the SLICC-SDI, total SLICC-SDI Score, ESR at inclusion, SELENA SLEDAI at inclusion, eGFR at inclusion, pericarditis at inclusion and proteinuria documented in the SLICC-SDI Score. Sex, age and total SLICC-SDI Score were significant (Table 4). Sex had an OR of 5.6 (95%-CI 2.3–13.7, p < 0.001). After inclusion of an interaction factor between sex and age, the effect of sex was less significant. However the overall model had a higher AIC compared to the model without the interaction factor. The table of estimates including the interaction factor is displayed in supplementary file 5. We performed an additional logistic regression model including cardiovascular risk factors such as hypertension, hyperlipidemia and diabetes mellitus type 2 (Table 5). Sex remained a significant risk factor for CAD.

**Table 4:** Logistic regression for coronary artery disease.

	Unadjusted model		Adjusted model		
	OR	95%-CI	OR	95%-CI	p-value
Sex	4.663	2.250–9.667	5.582	2.275–13.695	< 0.001
Age	1.060	1.036–1.085	1.038	1.010–1.066	0.007
Total SLICC-SDI score	1.620	1.417–1.852	1.598	1.391–1.834	< 0.001
Constant			0.002		< 0.001
Overall model					
AIC					169.727
p-value					< 0.001

Table of estimates of the logistic regression for coronary artery disease. Unadjusted model presents results from a univariate logistic regression, adjusted model presents results from the multivariate logistic regression including all shown variables. Sex (male = 1, female = 0), age at time of the SLICC-SDI Score and total SLICC-SDI Score were included in the model. OR = odds ratio, 95%-CI = 95%-confidence interval, AIC = akaike information criterion.

**Table 5:** Logistic regression for coronary artery disease including cardiovascular risk factors.

	OR	95%-CI	p-value
Gender	5.327	2.069–13.717	0.001
Age	1.030	1.001–1.060	0.044
Total SLICC-SDI Score	1.580	1.365–1.828	< 0.001
Hypertension	1.799	0.699–4.626	0.223
Diabetes mellitus type 2	0.809	0.219–2.994	0.751
Hyperlipidemia	3.933	1.299–11.904	0.015
Constant	0.002		< 0.001
Overall model			
AIC			165.763
p-value			< 0.001

Tables of estimates of multiple logistical regression models for coronary artery disease including cardiovascular risk factors. The model includes sex (male = 1, female = 0), age at time of the SLICC-SDI Score in years, total SLICC-SDI Score in points and presence of cardiovascular risk factors at inclusion to cohort such as hypertension, diabetes mellitus type 2 and hyperlipidemia. OR = odds ratio, 95%-CI = 95% confidence interval, AIC = Akaike information criterion.

We included sex, age at time of the SLICC-SDI, total SLICC-SDI Score, ESR at inclusion, eGFR at inclusion, chronic kidney disease (CKD) and proteinuria documented in the SLICC-SDI Score in the multiple logistic regression model for myocardial infarction. Sex, age, total SLICC SDI Score and CKD were significant (Table 6). Male sex had an OR of 8.3 (95%-CI 2.1–32.6, p = 0.002). After inclusion of the interaction factor between age and sex, the effect of sex was no longer significant. However, the model with the interaction factor had a higher AIC compared to the model without the interaction factor, suggesting its inferiority. The model including the interaction factor is displayed in supplementary file 7. An additional model including hypertension, hyperlipidemia and diabetes mellitus type 2 did not significantly change the impact of sex on myocardial infarction (Table 7).

**Table 6:** Logistic regression for myocardial infarction.

	Unadjusted model		Adjusted model		
	OR	95%-CI	OR	95%-CI	p-value
Sex	4.541	1.768–11.661	8.305	2.116–32.598	0.002
CKD	3.708	1.279–10.747	0.085	0.010—0.701	0.022
Age	1.070	1.038–1.104	1.050	1.004–1.099	0.033
Total SLICC-SDI Score	1.688	1.437–1.982	2.119	1.633–2.748	< 0.001
Constant			< 0.001		< 0.001
Overall model					
AIC					86.159
p-value					< 0.001

Table of estimates of the logistic regression model for myocardial infarction. Unadjusted model presents results from a univariate logistic regression, adjusted model presents results from the multivariate logistic regression including all shown variables. Sex (male = 1, female = 0), age in years at time of the SLICC-SDI Score, total SLICC-SDI Score and chronic kidney disease (CKD) documented in the SLICC-SDI Score were included in the model. OR = odds ratio, 95%-CI = 95%-confidence interval, AIC = Akaike information criterion.

**Table 7:** Logistic regression for myocardial infarction including cardiovascular risk factors.

	OR	95%-CI	p-value
Gender	4.770	1.070–21.259	0.040
CKD	0.116	0.014–0.954	0.045
Age	1.031	0.982–1.083	0.222
Total SLICC-SDI Score	2.061	1.582–2.685	< 0.001
Hypertension	2.371	0.557–10.096	0.243
Diabetes mellitus type 2	5.240	1.009–27.223	0.049
Hyperlipidemia	1.241	0.237–6.494	0.799
Constant	0.000		< 0.001
Overall model			
AIC			84.608
p-value			< 0.001

Tables of estimates of multiple logistical regression models for myocardial infarction including cardiovascular risk factors. The model includes sex (male = 1, female = 0), age at time of the SLICC-SDI Score in years, presence of chronic kidney disease, total SLICC-SDI Score in points and cardiovascular risk factors at inclusion to cohort such as hypertension, diabetes mellitus type 2, hyperlipidemia. OR = odds ratio, 95%-CI = 95% confidence interval, AIC = Akaike information criterion.

### Mortality

**Figure 2:**
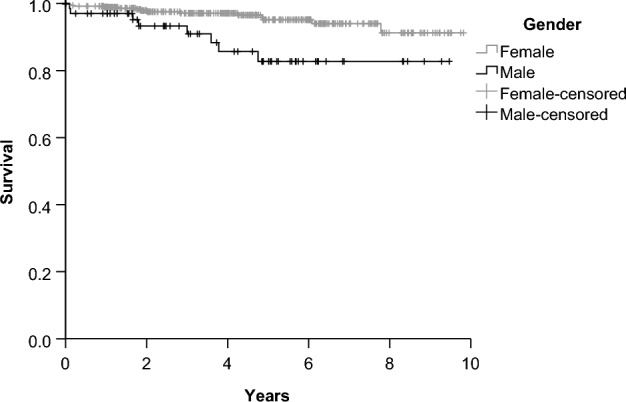
Kaplan Meier curve for overall mortality of male and female patients among study population.

In total 22 (4%) patients died during the follow up period of which 14 (3%) were women and 8 (9%) were men. The deaths of 6 patients were related to SLE. The causes of death in the other patients were infection, cerebrovascular accidents, end stage renal disease, heart insufficiency, brainstem vasculitis and in one case myocardial rupture due to bacterial infection and myocardial infarction. Kaplan Meier analysis showed a significantly worse survival in men with a p-value of 0.005. Men had an estimated mean survival time of 8.3 years and women 9.4 years. After 5 years estimated cumulative survival was 83% for men and 95% for women (Fig. 2). The age adjusted mortality hazards ratio between sexes was not significantly increased (hazard ratio 2.0 for male gender, 95%-CI 0.9–5.0).

## Discussion

We analyzed sex differences in 529 women and 93 men of the observational, prospective Swiss systemic lupus erythematosus cohort study and found significant differences between male and female SLE regarding clinical manifestations and renal and cardiovascular outcome.

We found a significantly higher risk in males for cardiovascular complications such as myocardial infarction with an OR of 8.3 and CAD with an OR of 5.6. Age was as well a significant risk factor for cardiovascular outcome. Our multivariate models suggests that an age-dependent sex difference may exist and explain partially the sex difference in cardiovascular outcome. Nevertheless, adding the interaction factor between sex and age did not improve the multivariate model. The model without the interaction factor was better by comparison of the AIC and showed a significant association between sex and CAD and myocardial infarction. This suggests that sex could be a risk factor for cardiovascular outcome independently of age. Similarly, in the mentioned LUMINA study men had a higher risk for any cardiovascular damage compared to women with an OR of 3.6^7^. This is in line with several other studies on SLE in males^8,22–24^.

It is known that in general population men have a higher cardiovascular risk than women^25^, this must have partly contributed to our findings that men had higher risk of CAD and myocardial infarction. The American heart association reported that women and men aged between 40 and 59 years had a prevalence of 1.8% versus 3.3% for myocardial infarction and 5.9% versus 6.3% for coronary heart disease^26^. Our observed risk difference between sexes in patients with SLE seems to be higher than the reported risk difference in general population, suggesting that the increased cardiovascular risk in men of the general population cannot solely explain our observed cardiovascular risk differences. Furthermore, after including traditional cardiovascular risk factors in our multivariate models for CAD and myocardial infarction, sex remained a significant risk factor.

Previous studies suggest a higher risk for patients with SLE to have any cardiovascular disease which can not only be explained by traditional cardiovascular risk factors^6^. Non traditional risk factors seem to have as well a big impact such as systemic inflammation, systemic disease and medication related risk factors^6,7^. We recently showed that serum calcification propensity measured by the T50 score test was closely associated with disease activity, suggesting that non traditional, lupus-specific risk factors contribute considerably to premature atherosclerosis and therefore cardiovascular events ^27^.

We found a worse renal outcome with a higher hazard ratio for renal failure in men than in women which is consistent with the large US-cohort of Tan et al. and other smaller cohorts who found as well significantly higher rates of renal insufficiency and renal failure in men^8,22,28^. In contrast, no differences were found in the rate of renal failure both in a recent study of a large low income US-population with lupus nephritis^29^, as well as in a rather small cohort with 30-years follow^10^. Differences in ethnicity, sample size, socioeconomic status, follow up period and definitions of renal failure may explain these controversial results. In the review by Murphy et al. it was suggested that these differences may be biased by the recruitment process, showing more renal involvement in studies held in nephrology clinics^9^. In our study however, patients were recruited in different specialty clinics which may avoid the specialty-based recruitment bias. Nevertheless, the multivariate model including cardiovascular risk factors showed no significantly increased hazard ratio for renal failure in men. This finding suggests that sex is not a sole risk factor for renal failure, but to some degree dependent on additional cardiovascular risk factors.

The age at diagnosis in this cohort was significantly higher in men than women which is consistent with several previous studies^8,22,30,31^. Population based studies in France and Germany showed that the incidence rates have a peak in a much younger age in women than in men^32,33^. In other studies however, the age at diagnosis was similar between the sexes^5,10,23,34^. This controversy could be due to ethnic and geographic factors which differ among the studies, since previously reported data seems to show higher age in European men^35^.

We were expecting a longer delay from first disease manifestation to SLE diagnosis in men, possibly linked to its rarity and postulated different clinical manifestation which would lead to a delayed consideration of SLE in the diagnostic process of these patients^36^. However, in our cohort time to diagnosis was similar between sexes. Therefore, a delay in diagnosis in men may not explain a possible greater burden of disease and damage. In a Latin American cohort the time to diagnosis was even significantly shorter in men, suggesting a faster progression to overt SLE^23^.

Regarding clinical manifestation of SLE, men were less likely to develop dermatological manifestations, arthritis and psychosis. In the multivariate models, female sex remained a risk factor for development of photosensitivity, any dermatological manifestations and arthritis. There was an interaction between disease duration and sex in the multivariate model for dermatological manifestations. However, in the multivariate model for arthritis and photosensitivity male sex remained unchanged and significantly associated with arthritis or photosensitivity regardless of the inclusion of the interaction factor with disease duration. A multivariate analysis for psychosis was not performed due to the very small number of patients, especially in men. Our results are in line with literature where men are less likely to have skin involvement^8,9,23,35^. Our findings regarding arthritis are consistent with multiple studies, including the Latin American cohort^23,35,37^. Other studies, however, showed no differences in arthritis^8,10,30,31,34^, but two of them found a higher prevalence of arthralgia in women^8,34^. Generally, our study suggests that men have the same spectrum of disease manifestations, but a possibly a difference in prevalence of certain manifestations than women.

The prevalence of renal involvement in men remains controversial ^9^. A higher prevalence of renal involvement was described in some studies, including the large cohort by Tan et al.^8,23,31,34^. Our study did not show a higher prevalence of renal involvement in men, which is consistent with a small Spanish^30^ and large multi-ethnic US cohort^5^.

We did not find any significant difference between the sexes in immunological manifestations. In contrast, a recent meta-analysis showed significantly higher prevalence of anti-dsDNA in men, higher prevalence of lupus anticoagulant and ANA in women and lower levels of complement factor 3 (C3) in women^35^. However, similar to our results the LUMINA study found no significant differences in immunological manifestations besides a higher prevalence of lupus anticoagulant in women^5^. The above mentioned Latin American cohort found no differences apart from significantly higher prevalence of Anti-cardiolipin antibodies and low C3 levels in men^23^.

Regardless the sex difference in cardiovascular damage, the overall damage measured by SLICC-SDI Score was similar between sexes which is in line with previous studies^23^. There was no difference in disease activity at inclusion as well.

The mortality rate was significantly higher in men than women in this cohort. In contrast, age adjusted hazard ratio for mortality was not significantly increased in men and women. The 1981 study of Wallace et al. and more recent studies as well^4,36^ reported a significant difference in mortality. We assume the number of deaths in our cohort was too small to adequately assess differences in mortality between sexes beyond the increased mortality hazard ratio of age, since there was an age difference between sexes.

Our work is the first sex study in a western European population. To date the larger studies that have examined sex differences have only a small proportion of Caucasians, and their results are therefore poorly generalizable to western European populations. Data of SLE from France show a prevalence of 47/100,000^33^, which means our cohort of 622 patients would account for approximately 15% of all SLE patients in Switzerland. The physicians recruiting patients for the cohort come from various disciplines, including dermatology, rheumatology, nephrology, immunology and internal medicine. In addition, these physicians do not all practice in a university hospital, but also in smaller regional hospitals and a private practice. This contributes significantly to the representativeness of our study. Furthermore, considering the impact of ethnicity in SLE disease course^12^, our results provide information for clinicians in other western European populations of which other SLE cohorts reported similar ethnical background^13^.

Our study has some limitations. The patients in our cohort have been included at different times of their disease course, some of them right after diagnosis, others after a long disease duration. This can influence the longitudinal findings. The age between the two compared groups was significantly different at baseline, which can confound the data as well. To counteract this, we included age and disease duration in our multivariate models for SLE manifestations, cardiovascular and renal outcomes. As in all cohort studies we cannot control our results for unknown or unmeasured confounders. Furthermore, since most patients had only once an evaluation of the SLICC-SDI Score, it allowed us only to uphold cross sectional information of cardiovascular events and overall damage.

The reasons for the observed sex differences in SLE-related outcomes are still unknown. Multiple reasonable hypotheses exist such as hormonal, sex chromosomal theories and intrauterine selection, but none of them achieve to fully explain the observed differences^36^. For example, sex hormonal hypothesis is supported by murine models where female hormones seem to increase risk of SLE and disease flares, while androgens seem to be beneficial. However, clinical trials could not confirm completely these effects on SLE in humans^36^. The sex chromosome theory is based on the finding that Klinefelter’s syndrome has a strong association with SLE, indicating that two X-chromosomes increase the risk of lupus by tenfold^36^. However, further investigations are needed to understand this association. Additionally, it has been proposed that behavioral factors have an influence as well, leading to lower likelihood of men with a mild disease to consult a doctor than women which can cause a statistical bias^23^.

## Conclusion

Our study investigated sex differences in SLE in a large national cohort and found significant differences. This was the first of its kind in a western European population. In our study, men were less likely to have arthritis and dermatological manifestations, especially photosensitivity than women. Regarding outcome, they had a higher risk for renal failure and male sex was a significant risk factor for cardiovascular events. Further research in this area is needed and could lead to a better understanding of the etiology of SLE in general and help provide sex specific treatment options.

### Supplementary Information

Supplementary Table 1.

Supplementary Table 2.

Supplementary Table 3.

Supplementary Table 4.

Supplementary Table 5.

Supplementary Table 6.

Supplementary Table 7.

Supplementary Information.

## Data Availability

The data that support the findings of this study are available from the Swiss SLE Cohort Study but are not publicly available. Data are however available from the authors upon reasonable request and with permission of the Swiss SLE Cohort Study.

## Abbreviations

SLE: Systemic lupus erythematosus
SLICC-SDI: Systemic Lupus International Collaborating Clinics/American College of Rheumatology Damage Index
CAD: Coronary artery disease
ESRD: End stage renal disease
ESR: Erythrocyte sedimentation rate
ANA: Anti-nuclear antibodies
Anti-dsDNA: Anti-double stranded DNA
SSCS: Swiss SLE Cohort Study
ACR: American College of Rheumatology
ISN/RPS: International Society of Nephrology and Renal Pathology Society
WHO: World Health Organization
SELENA SLEDAI: Systemic Lupus Erythematosus Disease Activity Index score with the Safety of estrogens in lupus erythematosus modification
eGFR: Estimated glomerular filtration rate
CKD-EPI: Chronic kidney disease epidemiology collaboration equation
CKD: Chronic kidney disease
SD: Standard deviation
SE: Standard error
PGA: Physicians global assessment
C3: Complement factor 3
95%-CI: 95% Confidence interval
AIC: Akaike information criterion
OR: Odds ratio
