# Supplementary material for: Worse cardiovascular and renal outcome in male SLE patients
Source: Sci Rep. 2023 Oct 30;13:18628. doi: 10.1038/s41598-023-45171-7 (PMC10616173; doi:10.1038/s41598-023-45171-7)
Supplement: Supplementary file 1 — Supplementary Table 1. [file 41598_2023_45171_MOESM1_ESM.docx]

Supplementary Table 1: SLE manifestations prior to inclusion according to ACR criteria

|  | Women | Men | p-value^a^ |
| --- | --- | --- | --- |
| Number of total ACR criteria (points) | 5 (4, 6) | 4 (4, 6) | **0.007** |
| Clinical manifestations |  |  |  |
| Dermatological disorder | 403 (76%) | 57 (61%) | **0.005** |
| Photosensitivity | 269 (51%) | 26 (28%) | **<0.001** |
| Malar rash | 208 (39%) | 28 (30%) | 0.105 |
| Discoid rash | 112 (21%) | 14 (15%) | 0.208 |
| Oral and nasal ulcers | 158 (30%) | 21 (23%) | 0.172 |
| Arthritis | 392 (74%) | 53 (57%) | **0.001** |
| Serositis | 142 (27%) | 32 (34%) | 0.168 |
| Pleuritis | 107 (20%) | 26 (28%) | 0.102 |
| Pericarditis | 96 (18%) | 16 (17%) | 0.885 |
| Renal disorder | 197 (37%) | 39 (42%) | 0.419 |
| Haematologic disorder | 317 (60%) | 60 (64%) | 0.490 |
| Central nervous system involvement | 46 (9%) | 7 (8%) | 0.842 |
| Psychosis | 34 (6%) | 1 (1%) | **0.047** |
| Seizures | 22 (4%) | 6 (6%) | 0.290 |
| Immunological manifestations |  |  |  |
| Anti-nuclear antibodies | 516 (98%) | 88 (95%) | 0.076 |
| Anti-dsDNA antibodies | 338 (64%) | 64 (69%) | 0.412 |
| Anti-Sm antibodies | 109 (21%) | 15 (16%) | 0.397 |
| Antiphospholipid antibodies | 231 (44%) | 34 (37%) | 0.210 |

Values were presented as medians with 25% and 75% quartile in brackets for continuous variables or as absolute values with percentages for categorical variables.

^a^ p-values<0.05 were considered statistically significant and written in bold.
