# Supplementary material for: Worse cardiovascular and renal outcome in male SLE patients
Source: Sci Rep. 2023 Oct 30;13:18628. doi: 10.1038/s41598-023-45171-7 (PMC10616173; doi:10.1038/s41598-023-45171-7)
Supplement: Supplementary file 2 — Supplementary Table 2. [file 41598_2023_45171_MOESM2_ESM.docx]

Supplementary table 2: logistic regression for arthritis

|  | Model 1 | | | Model 2 | | |
| --- | --- | --- | --- | --- | --- | --- |
|  | OR | 95%-CI | p-value | OR | 95%-CI | p-value |
| Gender | 0.474 | 0.290 – 0.775 | 0.003 | 0.308 | 0.162 – 0.585 | <0.001 |
| Anti-sm antibodies | 2.138 | 1.208 – 3.785 | 0.009 | 2.136 | 1.202 – 3.795 | 0.010 |
| Corticosteroids | 1.955 | 1.317 – 2.900 | 0.001 | 1.994 | 1.340 – 2.968 | 0.001 |
| eGFR | 1.008 | 1.001 – 1.015 | 0.022 | 1.008 | 1.001 – 1.015 | 0.021 |
| Disease duration | 1.045 | 1.017 – 1.073 | 0.001 | 1.035 | 1.008 – 1.03 | 0.012 |
| Gender * disease duration |  |  |  | 1.103 | 0.998 – 1.219 | 0.054 |
| Constant | 0.632 |  | 0.233 | 0.657 |  | 0.279 |
|  |  |  |  |  |  |  |
| overall model |  |  |  |  |  |  |
| AIC |  |  | 623.96 |  |  | 621.49 |
| p-value |  |  | <0.001 |  |  | <0.001 |

Tables of estimates of multiple logistical regression models for arthritis. Model 1 includes sex (male=1, female=0), anti-Sm antibodies at inclusion, use of oral corticosteroids at inclusion, eGFR at inclusion and disease duration at inclusion in years. Model 2 includes additionally the interaction factor between sex and disease duration. OR = odds ratio, 95%-CI = 95% confidence interval, AIC = akaike information criterion.
