# Supplementary material for: Worse cardiovascular and renal outcome in male SLE patients
Source: Sci Rep. 2023 Oct 30;13:18628. doi: 10.1038/s41598-023-45171-7 (PMC10616173; doi:10.1038/s41598-023-45171-7)
Supplement: Supplementary file 3 — Supplementary Table 3. [file 41598_2023_45171_MOESM3_ESM.docx]

Supplementary table 3: logistic regression for any dermatological manifestations of SLE

|  | OR | 95%-CI | p-value |
| --- | --- | --- | --- |
| Gender | 0.510 | 0.318 – 0.819 | 0.005 |
| disease duration | 1.022 | 0.998 – 1.047 | 0.072 |
| constant | 2.645 |  | <0.001 |

Tables of estimates of multiple logistical regression models for dermatological manifestations of SLE. The model includes sex (male=1, female=0) and disease duration at inclusion in years. OR = odds ratio, 95%-CI = 95% confidence interval.
