# Supplementary material for: Worse cardiovascular and renal outcome in male SLE patients
Source: Sci Rep. 2023 Oct 30;13:18628. doi: 10.1038/s41598-023-45171-7 (PMC10616173; doi:10.1038/s41598-023-45171-7)
Supplement: Supplementary file 4 — Supplementary Table 4. [file 41598_2023_45171_MOESM4_ESM.docx]

Supplementary table 4: logistic regression for photosensitivity

|  | OR | 95%-CI | p-value |
| --- | --- | --- | --- |
| Gender | 0.409 | 0.241 – 0.696 | 0.001 |
| Ethnic background |  |  | 0.033 |
| Ethnic background (1) | 0.000 |  | 1.000 |
| Ethnic background (2) | 0.202 |  | 1.000 |
| Ethnic background (3) | 0.184 |  | 1.000 |
| Ethnic background (4) | 0.000 |  | 0.999 |
| Ethnic background (5) | 0.000 |  | 1.000 |
| Anti dsDNA antibodies | 0.489 | 0.333 – 0.718 | <0.0001 |
| PGA | 1.389 | 1.108 – 1.740 | 0.004 |
| Antimalarial medication | 1.711 | 1.153 – 2.540 | 0.008 |
| Disease duration | 1.043 | 1.020 – 1.068 | <0.0001 |
| Constant | 0.000 |  | 0.999 |

Tables of estimates of multiple logistical regression models for photosensitivity. The model includes sex (male=1, female=0), ethnic background, anti-dsDNA antibodies at inclusion, PGA Score at inclusion, use of antimalarial medication at inclusion and disease duration at inclusion in years. OR = odds ratio, 95%-CI = 95% confidence interval.
