# Supplementary material for: Worse cardiovascular and renal outcome in male SLE patients
Source: Sci Rep. 2023 Oct 30;13:18628. doi: 10.1038/s41598-023-45171-7 (PMC10616173; doi:10.1038/s41598-023-45171-7)
Supplement: Supplementary file 5 — Supplementary Table 5. [file 41598_2023_45171_MOESM5_ESM.docx]

Supplementary table 5: cox regression model for renal outcome with cardiovascular risk factors

|  | HR | 95%-CI | p-value |
| --- | --- | --- | --- |
| Sex | 1.640 | 0.746 – 3.607 | 0.219 |
| eGFR at inclusion | 0.962 | 0.951 – 0.973 | <0.001 |
| Hypertension | 1.854 | 0.888 – 3.871 | 0.100 |
| Diabetes mellitus type 2 | 0.752 | 0.214 – 2.645 | 0.657 |
| Hyperlipidemia | 0.592 | 0.189 – 1.849 | 0.367 |
| Coronary artery disease | 1.384 | 0.515 – 3.721 | 0.519 |
|  |  |  |  |
| Overall model |  |  |  |
| AIC | 402.308 | | |
| p-value | <0.001 | | |

Tables of estimates of cox regression models for renal failure including cardiovascular risk factors. Model includes sex (male=1, female=0), eGFR = estimated glomerular filtration rate at inclusion to cohort, presence of hypertension, diabetes mellitus type 2, hyperlipidemia and coronary artery disease at inclusion to cohort. HR = hazard ratio, 95%-CI = 95% confidence interval, AIC = akaike information criterion.
