# Supplementary material for: Worse cardiovascular and renal outcome in male SLE patients
Source: Sci Rep. 2023 Oct 30;13:18628. doi: 10.1038/s41598-023-45171-7 (PMC10616173; doi:10.1038/s41598-023-45171-7)
Supplement: Supplementary file 6 — Supplementary Table 6. [file 41598_2023_45171_MOESM6_ESM.docx]

Supplementary table 6: logistic regression for coronary artery disease

|  | Model 1 | | | Model 2 | | |
| --- | --- | --- | --- | --- | --- | --- |
|  | OR | 95%-CI | p-value | OR | 95%-CI | p-value |
| Gender | 5.582 | 2.275 – 13.695 | <0.001 | 31.186 | 1.009 - 964.061 | 0.049 |
| Age | 1.038 | 1.010 – 1.066 | 0.007 | 1.048 | 1.014 – 1.083 | 0.006 |
| Total SLICC-SDI Score | 1.598 | 1.391 – 1.834 | <0.001 | 1.614 | 1.400 – 1.860 | <0.001 |
| Gender * Age |  |  |  | 0.972 | 0.921 – 1.026 | 0.311 |
| Constant | 0.002 |  | <0.001 | 0.001 |  | <0.001 |
|  |  |  |  |  |  |  |
| overall model |  |  |  |  |  |  |
| AIC |  |  | 169.727 |  |  | 170.729 |
| p-value |  |  | <0.001 |  |  | <0.001 |

Tables of estimates of multiple logistical regression models for coronary artery disease. Model 1 includes sex (male=1, female=0), age at time of the SLICC-SDI Score in years, total SLICC-SDI Score in points. Model 2 includes additionally the interaction factor between sex and age. OR = odds ratio, 95%-CI = 95% confidence interval, AIC = akaike information criterion.
