# Supplementary material for: Worse cardiovascular and renal outcome in male SLE patients
Source: Sci Rep. 2023 Oct 30;13:18628. doi: 10.1038/s41598-023-45171-7 (PMC10616173; doi:10.1038/s41598-023-45171-7)
Supplement: Supplementary file 7 — Supplementary Table 7. [file 41598_2023_45171_MOESM7_ESM.docx]

Supplementary table 7: logistic regression for myocardial infarction

|  | Model 1 | | | Model 2 | | |
| --- | --- | --- | --- | --- | --- | --- |
|  | OR | 95%-CI | p-value | OR | 95%-CI | p-value |
| Gender | 8.305 | 2.116 – 32.598 | 0.002 | 5.073 | 0.006 – 4246.349 | 0.636 |
| CKD | 0.085 | 0.010 – 0.701 | 0.022 | 0.086 | 0.010 – 0.709 | 0.023 |
| Age | 1.050 | 1.004 – 1.099 | 0.033 | 1.048 | 0.993 – 1.106 | 0.091 |
| Total SLICC-SDI Score | 2.119 | 1.633 – 2.748 | <0.001 | 2.111 | 1.622 – 2.746 | <0.001 |
| Gender * Age |  |  |  | 1.008 | 0.912 – 1.114 | 0.883 |
| Constant | 0.000 |  | <0.001 | 0.000 |  | <0.001 |
|  |  |  |  |  |  |  |
| overall model |  |  |  |  |  |  |
| AIC |  |  | 86.159 |  |  | 88.137 |
| p-value |  |  | <0.001 |  |  | <0.001 |

Tables of estimates of multiple logistical regression models for myocardial infarction. Model 1 includes sex (male=1, female=0), age at time of the SLICC-SDI Score in years, presence of chronic kidney disease, total SLICC-SDI Score in points. Model 2 includes additionally the interaction factor between sex and age. OR = odds ratio, 95%-CI = 95% confidence interval, AIC = akaike information criterion.
