# Supplementary material for: Worse cardiovascular and renal outcome in male SLE patients
Source: Sci Rep. 2023 Oct 30;13:18628. doi: 10.1038/s41598-023-45171-7 (PMC10616173; doi:10.1038/s41598-023-45171-7)
Supplement: Supplementary file 8 — Supplementary Information. [file 41598_2023_45171_MOESM8_ESM.docx]

Members of the The Swiss Systemic Lupus Erythematosus Cohort Study Group (SSCS)

| Chizzolini Carlo | Pathology and Immunology, Centre Médical Universitaire, School of Medicine, Geneva University, Switzerland |
| --- | --- |
| Comte Denis | Service of Immunology and Allergology, Department of Medicine, University Hospital Lausanne, Lausanne, Switzerland. |
| Dahdal Suzan | Department of Nephrology and Hypertension lnselspital, University Hospital, Bern, Switzerland. |
| Eisenberger Ute | Department of Nephrology, University Hospital Essen, Essen, Germany. |
| Hauser Thomas | IZZ Immunologie-Zentrum Zürich, Zürich, Switzerland. |
| Huynh-Do Uyen | Department of Nephrology and Hypertension lnselspital, University Hospital, Bern, Switzerland. |
| Ribi Camillo | Division of Clinical Immunology and Allergy, University Hospital Lausanne, Lausanne, Switzerland. |
| Roux-Lombard Pascale | Department of Immunology and Allergy, Geneva University Hospital, Geneva, Switzerland. |
| Rubbert-Roth Andrea | Department of Dermatology, Cantonal Hospital St, Gallen St, Gallen Switzerland. |
| Steiner Urs | Department of Immunology, University Hospital of Zurich, Zurich, Switzerland. |
| Trendelenburg Marten | Division of Internal Medicine and Clinical Immunology Laboratory, Department of Biomedicine, University Hospital Basel, Basel, Switzerland. |
| von Kempis Johannes | Division of Rheumatology and Immunology, Department of Internal Medicine, Kantonsspital St Gallen, St Gallen, Switzerland. |

Nominated consortia representative: Trendelenburg Marten, [marten.trendelenburg@usb.ch](mailto:marten.trendelenburg@usb.ch)
